# Loss of GABAergic cortical neurons underlies the neuropathology of Lafora disease

**DOI:** 10.1186/1756-6606-7-7

**Published:** 2014-01-28

**Authors:** Saida Ortolano, Irene Vieitez, Roberto Carlos Agis-Balboa, Carlos Spuch

**Affiliations:** 1 Group of Rare Diseases, Institute of Biomedical Research of Vigo (IBIV), Xerencia de Xestion Integrada de Vigo, SERGAS, Psychiatric Hospital Rebullón, Puxeiros s/n, Pontevedra 36415 Mos, Spain; 2 Group of Neurodegenerative Diseases and Psychiatric Disorders, Institute of Biomedical Research of Vigo (IBIV), Xerencia de Xestion Integrada de Vigo, SERGAS, Psychiatric Hospital Rebullón, Puxeiros s/n, Pontevedra 36415 Mos, Spain

**Keywords:** BDNF, Cerebral cortex, GABAergic neurons, Lafora disease, Laforin, Neurotrophin, NGF, p75NTR

## Abstract

**Background:**

Lafora disease is an autosomal recessive form of progressive myoclonic epilepsy caused by defects in the *EPM2A* and *EPM2B* genes. Primary symptoms of the pathology include seizures, ataxia, myoclonus, and progressive development of severe dementia. Lafora disease can be caused by defects in the *EPM2A* gene, which encodes the laforin protein phosphatase, or in the *NHLRC1* gene (also called *EPM2B*) codifying the malin E3 ubiquitin ligase. Studies on cellular models showed that laforin and malin interact and operate as a functional complex apparently regulating cellular functions such as glycogen metabolism, cellular stress response, and the proteolytic processes. However, the pathogenesis and the molecular mechanism of the disease, which imply either laforin or malin are poorly understood. Thus, the aim of our study is to elucidate the molecular mechanism of the pathology by characterizing cerebral cortex neurodegeneration in the well accepted murine model of Lafora disease *EPM2A*-/- mouse.

**Results:**

In this article, we want to asses the primary cause of the neurodegeneration in Lafora disease by studying GABAergic neurons in the cerebral cortex. We showed that the majority of Lafora bodies are specifically located in GABAergic neurons of the cerebral cortex of 3 months-old *EPM2A*-/- mice. Moreover, GABAergic neurons in the cerebral cortex of younger mice (1 month-old) are decreased in number and present altered neurotrophins and p75NTR signalling.

**Conclusions:**

Here, we concluded that there is impairment in GABAergic neurons neurodevelopment in the cerebral cortex, which occurs prior to the formation of Lafora bodies in the cytoplasm. The dysregulation of cerebral cortex development may contribute to Lafora disease pathogenesis.

## Background

Lafora disease (OMIM 254780) is an autosomal recessive form of epilepsy with onset in late childhood or adolescence [1-3]. Primary symptoms include seizures, ataxia, myoclonus, and the progressive development of severe dementia. Most affected individuals die by 25 years of age. Currently, there is no long-term treatment available. Clinically, Lafora disease is characterized by the presence of inclusion bodies, called Lafora bodies (LBs), which are present in several organs, including brain, heart, liver, muscle, and skin. LBs contain around 6% of protein and 90% of a poorly branched form of glycogen, which resembles amylopectin [4], suggesting that in Lafora disease, enzymes involved in glycogen metabolism are dysregulated.

The great majority of mutations causing Lafora disease have been identified in two genes: *EPM2A* (encoding laforin) a member of the dual-specificity protein phosphatase family [5,6], and *NHLRC1* (encoding malin) a protein with an NH2-terminal RING finger domain, characteristic of an important group of E3-ubiquitin ligases [7,8]. There is also evidence for the existence of a third locus [9], which was recently mapped in position 4q21.21, where is located the PRDM8, a gene of unknown function [10]. Patients carrying homozygous mutations in laforin or malin are phenotypically indistinguishable, suggesting that both proteins contribute to the same physiological pathway. LBs are localized in cell cytoplasm in several organs of affected patients. Although LBs are the hallmark of the disease, it is still unclear whether they are the cause of the pathology or are a simple consequence of this condition in the brain. LBs presence appears to be restricted to neurons, however in 2011 Valles-Ortega et al. described the accumulation of polyglucosan in the astrocytes of malin-KO mice [11]. Polyglucosan inclusions gradually replace the cytoplasms in a large number of dendrites, likely underlying onset and progression of this disease. The LBs are also marked by anti-ubiquitin antibodies, suggesting the accumulation of unmetabolized proteins in the bodies [12]. Therefore, Lafora disease may be a disorder of both carbohydrate metabolism and protein clearance [13].

Laforin is a member of the atypical dual specificity protein phosphatase family [5], which additionally contains a functional CBM20 carbohydrate-binding module. Two different, not mutually exclusive, roles have been suggested for laforin. First, laforin could act as a glycogen phosphatase, removing phosphatases from glycogen and preventing the soluble glycogen molecules from becoming insoluble polyglucosan [14-16]. On the other end, laforin interacts with the E3 ubiquitin ligase, malin, driving specific substrates related to glycogen metabolism in proximity of malin to be polyubiquitinated and degraded by the proteosome complex [17,18]. Experiments by Roach proposed that laforin is a physiological glycogen phosphatase whose impairment leads to the structural abnormalities in glycogen and to LB formation [14,16].

Several transgenic mouse models have been developed for Lafora disease, either by disruptiing *EPM2A* gene (null mice) [12] or by over-expressing inactivated laforin [19] in all tissues. A malin knock out mouse was also developed by disruption of the *NHLRC1* gene [16,20]. All these transgenic models mimic the human disease, since mice LBs are accumulated in cell cytosol and they develop epilepsy, however they differ from affected patients in terms of life span, which is not shortened in the animals.

The development of a Lafora like phenotype in laforin knockout animals supports the hypothesis that laforin dephosphorylates glycogen *in vivo*[14]. Increased phosphorylation is associated with disturbances in glycogen structure that are consistent with LB formation [15]. LB accumulation was believed to coincide with increased neuronal non-apoptotic cell death, development of seizures in Lafora patients [12] and ultimately, for patient’s death [4]. Brain biopsies confirmed the accumulation of starch-like compound in neuron, overtaking dendrites, as a possible cause of disease and neurodegeneration. This hypothesis was corroborated by Minsassian [21] et al., which showed for the first time, that the removal of a protein targeting to glycogen (PTG) in an animal model of Lafora disease reduced LB formation and eliminates neuronal loss and the myoclonic epilepsy.

Despite the fact that the genetic defect in Lafora’s patients is present since birth, any molecular explanation for the disease must take into account the gradual nature of pathology onset, whose symptoms usually appear in teenage years [22]. A well accepted hypothesis states that symptoms of the disease are a consequence of LB accumulation, which in neurons leads to cell death and the associated epilepsy, myoclonus and ataxia. Nevertheless, considering that not all neurons undergoing cell death in the laforin ko mouse present visible LB, we propose that LB formation could be the final result of multiple aberrant steps in glycogen synthesis, and that earlier non-visible products are responsible for neuronal apoptosis. Based on this hypothesis, we provide evidence that prior to the development of the first LB there is a specific damage in neurodevelopment of GABAergic neurons in the cerebral cortex.

## Results

### Lafora bodies are specifically located in GABAergic cortical neurons at early age

We detected the presence of LBs in neurons of *EPM2A*-/- mice at 3 and 13 months-old by immunostaining of neuronal marker βIII-Tubulin, Periodic acid–Schiff (PAS) and KM279 polyglucosan immumoreactive antibody (Additional file 1: Figure S1). LBs are easy to localize by microscope, since light is refracted through the body. We combined βIII-Tubulin and PAS staining to detect LBs specifically located in neurons. It LBs, commonly distinguished by size and location, are compartmentalized to perikaryon and dendrites and not to axons. The bigger LBs are located in the perikaryal and the granular LBs are placed in the neuronal processes, mostly represented by dendrites [19,21].

To characterize the causes of neurodegeneration we evaluated PAS positives ground-glass inclusions, as well as LBs, in all brain regions of *EPM2A*-/- mice at the ages of 5 and 15 days and 1, 3, 5, 13 and 18 months. Although the LBs and ground-glass inclusions appear in all brain regions, the highest density was placed in cerebral cortex and hippocampus (Additional file 2: Figure S2), with lower amounts in the basal forebrain and random distribution throughout the rest of the brain. Ultrastructural analysis of the neuropile in the brain of *EPM2A*-/- mice revealed unequivocal features of somatic degeneration in cerebellar Purkinje cells (isolated or in full rows), hippocampal pyramidal and granular cells, and cerebral cortical pyramidal cells at early age (2 months-old mice) [12]. However, overtime evaluation of PAS positive inclusions that we performed revealed that the first LB in the cytoplasm appears 3 months old mice, while the PAS-positive ground-glass inclusions are present as early as at 1 month of age. Therefore we estimated that the time window for the iniciation of the neurodegenerative processes could be fixed between 15 days and 1 month of age. For this reason, we performed the additional molecular studies using *EPM2A*-/- in this range of time.

**Figure 1:**
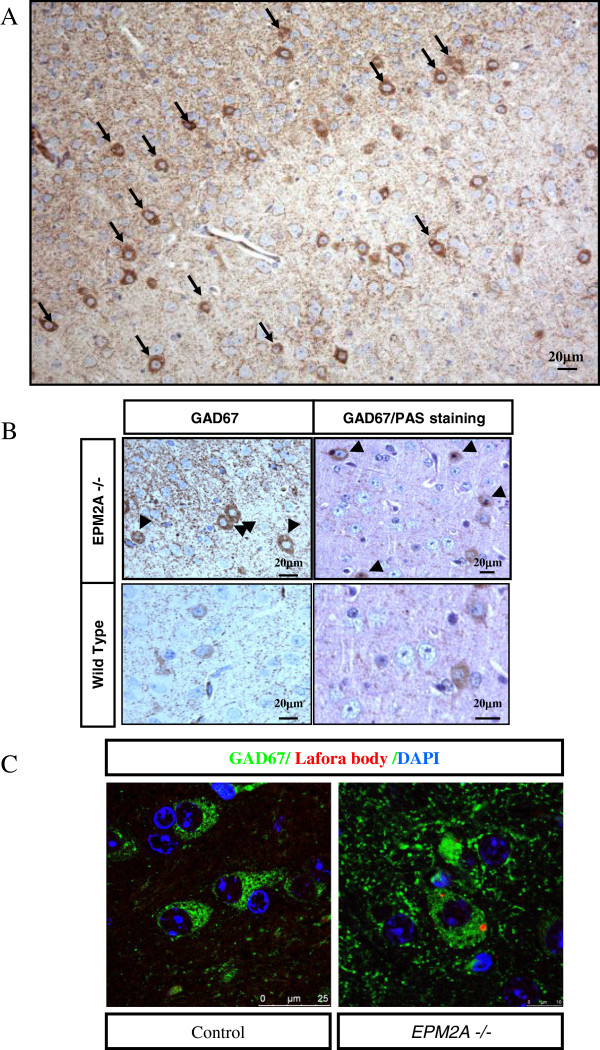
**LBs are specifically localized in GABAergic cortical neurons. A)** Broad image of the specific localization of LBs are only in GABAergic cortical neurons in paraffin sections of temporal cortex of *EPM2A*-/- mice (3 months-old) revealed with DAB. The cortical neuronal marker used in the immunostaining is the GAD67. **B)** Magnification of the picture 3A (left) of LBs located in the cytoplasm and combination with PAS staining (right). **C)** Immunofluorescence of GAD67 (green), polyglucosan antibody (red) and DAPI staining (blue) in wild type (left) and *EPM2A-*/- mice (right). These results showed that the specific localization of LBs exclusively in the GABAergic neurons at early ages gives us a new perspective about the sensible damage induced in these neurons due to laforin deficiency.

The first signal of the possible role of a dysfunction in cerebral cortex in Lafora disease came form the evidence that in 3 months-old *EPM2A*-/- all the LBs were specifically located in GABAergic neurons (GABAergic cells: so called because their neurotransmitter is gamma-aminobutyric acid or GABA) of the cerebral cortex (Figure 1), while LBs were located in all types of neurons in mice over 3 months old.

We also carried out immnostaining of GABAergic neurons with GAD67 marker in cerebral cortex, showing that all the LBs are placed in the GABAergic cortical neurons before 3 months (Figure 1A, LBs marked with arrows). GAD67 was previously used to identify GABAergic cortical neurons [23]. This result was confirmed with double staining of GAD67 positive neurons with either PAS (Figure 1B) or polyglucosan marker KM279 antibody (Figure 1C, LBs in red, GABAergic neurons in green).

### The population of GABAergic cortical neurons is reduced in Laforin-deficient mice

**Figure 2:**
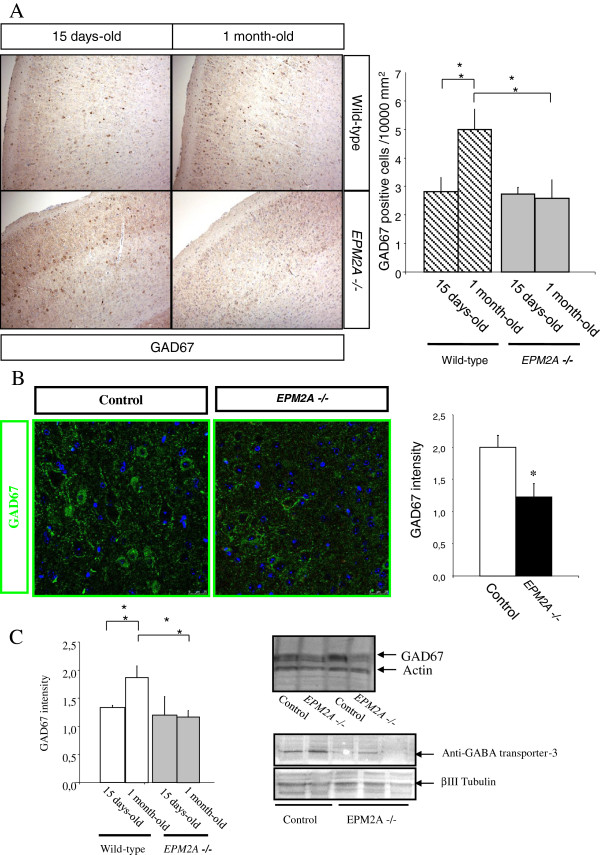
**Decrease of GABAergic neurons density in laforin-deficient mice.** We measured by different techniques the decrease of GABAergic cortical neurons in *EPM2A-*/- mice (1 month-old) (*P < 0.05). **A)** Immunohistochemistry performed in paraffin section of temporal cortex revealed with DAB, showing a strong reduction of GABAergic cortical neurons. Cell counting of GABAergic cortical neurons in 15 days-old and 1 month-old *EPM2A-*/- mice (n = 3) shows a significant reduction of 54.70% in the number of GAD67 neurons in *EPM2A*-/- compared with wild-type, which suggest the importance of the temporary window between 15 days and 1 month of age. **B)** Immunofluorescence and confocal study (n = 3) corroborating the same results. **C)** Cerebral cortex lysates measured by western blot (n = 5). This gel shows a clear GAD67 reduction in the cerebral cortex of 1 month old laforin-deficient mice.

We performed a comparative study of GABAergic neurons density in the cerebral cortex between *EPM2A*-/- and wild type mice at the age of 15 days-old and 1 month-old. The density of GAD67 positive neurons in the cortical layers was estimated by dividing the number of GAD67 neurons by the surface area in the cortical layer. We could not find any differences between groups (wild type vs. *EPM2A*-/-) until postnatal day 15 (P15). Following the analysis, we found that there was a significant reduction of (54.70%) in the number of GAD67 neurons in *EPM2A*-/- compared to wild-type mice (Figure 2A) at one month, coinciding with the appearance of the first LB in the cortical area of *EPM2A*-/- brain. We also found that at P30 GAD67 positive cell density increases in wild type mice compared to levels detected at P15, while it is maintained in *EPM2A*-/- mice, highlighting an impairment of GABAergic neurons development in the knockout mouse (Figure 2A). The comparative study was repeated by immunofluorescence, to quantify GAD67 fluorescence in confocal microscopy images using the Leica AMD morphometric software. We measured GAD67 intensity in random slides of cortical areas samples from 1 month-old wild-type and *EPM2A*-/- mice (Figure 2B) and find that GAD67 intensity was decreased of 61.20% (*p* < 0.05).

To further confirm our results, we measured GAD67 levels in brain cerebral cortex lysates from wild-type and *EPM2A*-/-, by western blot. Compatibly with previous data, we detected a significant reduction of GAD67 in *EPM2A*-/- mice at P30 (Figure 2C), since the neuronal population decrease at 47.60%. To exclude that the absence of laforin could interfere with GAD67 production, we repeated the western blot analysis using the Anti-GABA transporter-3 antibody (Millipore), obtaining results comparable to the previously described experiment (Figure 2C).

### Loss of synapses in the cerebral cortex of Laforin-deficient mice

**Figure 3:**
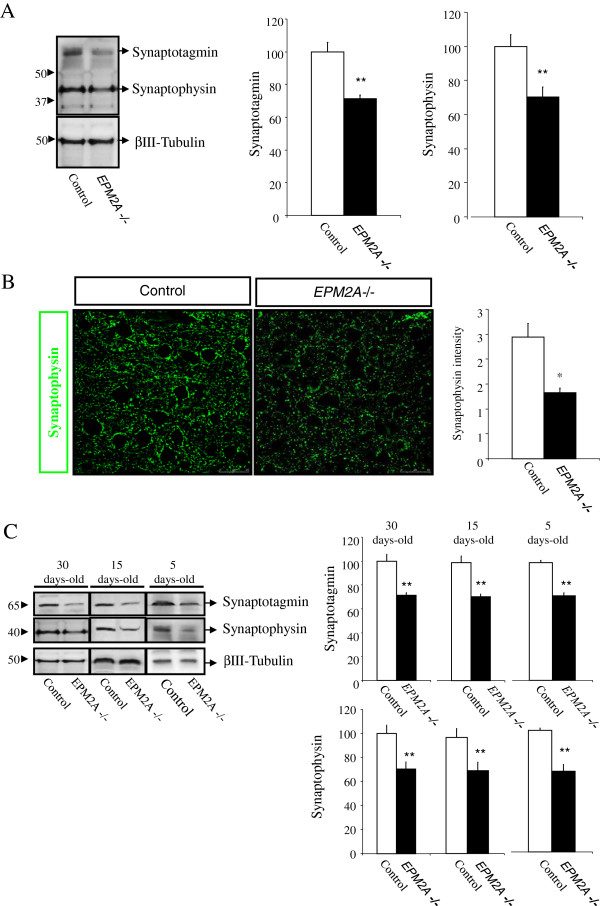
**Synaptic damage in the cerebral cortex of *EPM2A-*/- mice (1 month-old) measured by different techniques. A)** Representative western blot of quantification of two synaptic proteins (synaptophysin and synaptotagmin) in cerebral cortex lysates (n = 5). Comparable amounts of samples are loaded. This result demonstrated clearly the reduction synaptic proteins in the cerebral cortex of laforin-deficiency mice. **B)** Semi-quantification of synaptophysin measured by confocal microscopy in sections of cerebral cortex of *EPM2A*-/- mice (n = 3). Moreover, this experiment also revealed the reduction of synaptic protein in these mice. **C)** Western blot quantification of two synaptic proteins, synaptophysin and synaptotagmin, in cerebral cortex lysates of *EPM2A*-/- mice at different ages: 5, 15 and 30 days-old. These results illustrates that the synaptic damage occur at very early ages. Results are mean ± SEM *p < 0.05, **p < 0.01 wild-type mice vs *EPM2A* -/- mice (ANOVA followed by Student’s *t* test).

We analyzed the levels of synaptic protein in cerebral cortex on *EPM2A*-/- mice compared to wild-type. We performed western blot experiments using cortex cellular lysates from 1 month-old *EPM2A*-/- and wild-type mice. One month-old *EPM2A*-/- mice showed a dramatic decrease of immunoreactive levels of two synaptic proteins (synaptophysin and synaptotagmin) (Figure 3A). These results were confirmed through immunofluorescence and confocal microscope analysis; finding synaptophysin reduction in the cerebral cortex of *EPM2A*-/-mice (Figure 3B). To determine whether synaptic loss is due to LBs or to a lack of laforin, we measured both synaptic proteins (synaptophysin and synaptotagmin) in brain cerebral cortex at P5, P15 and P30 *EPM2A*-/- mice, finding that the levels of synaptic proteins were reduced at very early ages, starting at P5 *EPM2A*-/- mice (Figure 3C).

### Increase of lysosomal activity and actin disarrangement in cortical neurons of Laforin-deficient mice

**Figure 4:**
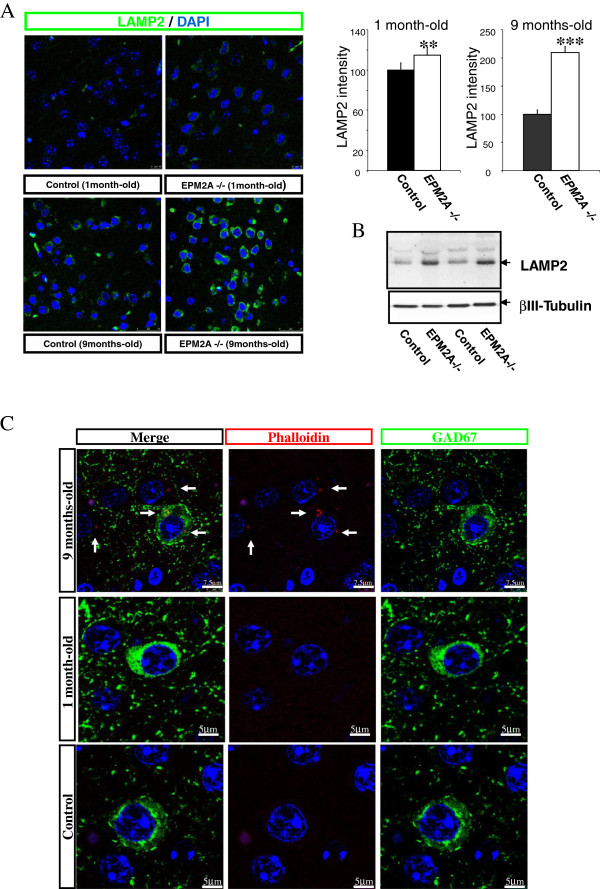
**Increase of lysosomal activity in the cerebral cortex of *EPM2A-*/- mice. A)** Confocal images of LAMP2 (green) and quantification of LAMP2 intensity in cerebral cortex of *EPM2A-*/-mice at 1 and 9 months of age (n = 5). Results are mean ± SEM ** p < 0.01 and *** p < 0.001 wild-type mice vs *EPM2A* -/- mice (ANOVA followed by Student’s *t* test). This quantification showed the increase of lysosomal activity in the cerebral cortex at late ages. **B)** Quantification of LAMP2 protein in cerebral cortex lsyates of *EPM2A*-/- mice (1 month-old, n = 5). Western blots represent the increase of the LAMP2 protein in the cerebral cortex lysates. **C)** F-actin accumulation in cerebral cortex of *EPM2A*-/- mice. Sections of cerebral cortex were stained with rhodamine-phalloidin. F-actin was detected in small clusters in the cell body of neurons in the cerebral cortex only in older laforin-deficient mice. The clusters of disarrangement of F-actin are pointed with white arrows in the representative picture.

We measured lysosomal activity with confocal microscopy and western blot experiments in cerebral cortex of *EPM2A*-/- at 1 and 9 months of age, compared with wild-type mice. Confocal microscopy, revealed a strong increase in LAMP2 intensity at 9 months (*p* < 0.001), however, we also found a slight increase of florescence signal at 1 month-old (*p* < 0.01) (Figure 4A). Western blot analysis confirmed an increased LAMP2 expression in cerebral cortex lysates from *EPM2A*-/- mice (Figure 4B).

To investigate whether the presence of LBs are implicated in the cytoskeletal organization, we stained cerebral cortex of *EPM2A*-/- at 1 and 9 months-old with the filamentous actin indicator rhodamine-phalloidin. The confocal images revealed a significant disarrangement of F-actin in brains of laforin-deficient mice but only at the age of 9 months. White arrows indicate cytoskeleton disarrangement in neurons cytoplasm [Figure 4C].

### Increase of caspase-3 activity in cortical neurons of Laforin-deficient mice

**Figure 5:**
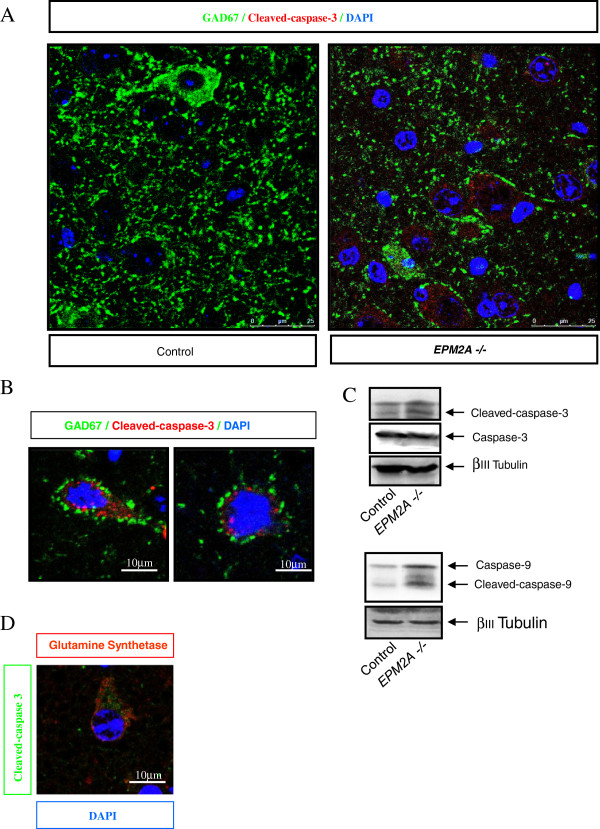
**Increase of caspase-3 activity in cortical neurons of *EPM2A-*/- mice. A)** Confocal images of cerebral cortex sections of *EPM2A*-/- mice with active-caspase-3 (red) and GAD67 (green). **B)** Magnification of the microphotography in panel **A**. These representative images revealed the activation of caspase-3 in non-GABAergic neurons. The GABAergic neurons contacts mainly with glutamatergic neurons and these neurons seem to activate the caspase-3 in laforin-deficient mice. **C)** Representative western blot of active-caspase-3 and active-caspase-9 quantification in the lysates of cerebral cortex in *EPM2A*-/- mice (1 month-old). **D)** Confocal images of cerebral cortex sections of *EPM2A*-/- mice with active-caspase-3 (green) and glutamatergic neuronal marker Glutamine synthetase Clone Gs-6 (red).

To study whether the lack of laforin induced a specific damage in GABAergic neurons we performed immunostaining and western blot for active-caspase-3 to detect apoptotic neurons in P5, P15 and P30 *EPM2A*-/- mice. We found an increase of active-caspase-3 in the cerebral cortex of 1 month-old *EPM2A*-/- mice (Figure 5), however we could not detect TUNEL-positive neurons (data not show). In some apoptotic pathways caspase-9 is upstream to caspase-3, thus, we checked caspase-9 activation in the same lysates, finding similar results (Figure 5C). Surprisingly by co-staining neurons with Glutamine synthetase Clone Gs-6 marker (red) and active-caspase-3 (green), we found that active-caspase-3 appeared only in the glutamatergic neurons of the cerebral cortex (Figure 5D).

### Laforin deficiency induces nuclear translocation of p53 in the cerebral cortex

**Figure 6:**
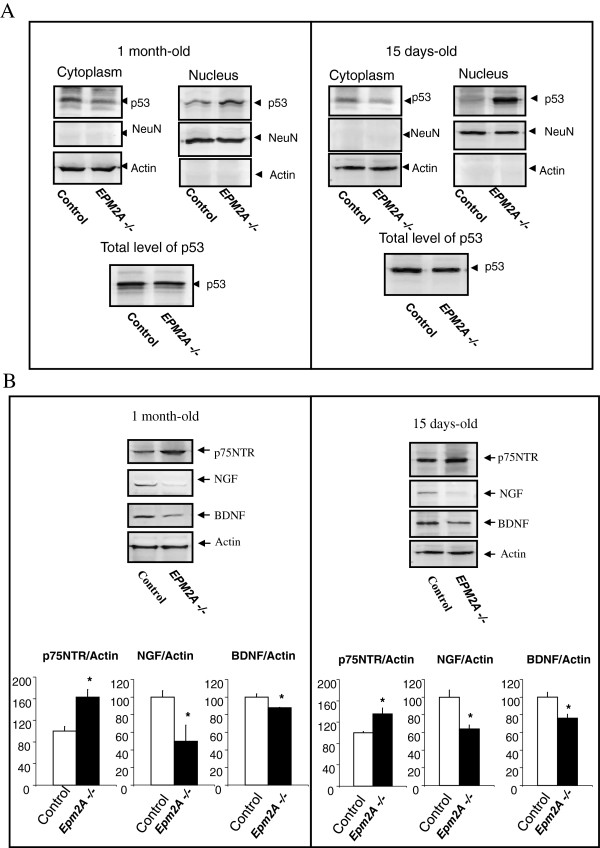
**Altered levels of neurotrophins and subcellular distribution of p53 in cerebral cortex of *EPM2A*-/- mice. A)** Subcellular distribution of p53 in cerebral cortex of *EPM2A*-/- mice. In samples of cerebral cortex of *EPM2A*-/- and wild type mice (n = 5) at different ages (15 and 30 days-old), we measured the content of p53 in the nuclear and cytoplasmic fractions by western blot. This picture illustrates the accumulation of p53 protein in the nucleus fraction of the cerebral cortex in Laforin-deficiency mice compared to wild-type. There is no change in the content of p53 in lysate of whole cerebral cortex at both ages. Results are mean ± SEM *p < 0.05, ** p < 0.01 wild-type mice vs *EPM2A* -/- mice (ANOVA followed by Student’s *t* test). **B)** To characterize the NGF and BDNF expression in cerebral cortex from *EPM2A*-/- mice, we quantified the levels of the neurotrophins ligands NGF and BDNF and the receptor implicated in the apoptosis of neurons, the p75NTR, in lysates of cerebral cortex by western blot analysis. This picture illustrate the decrease of NGF and BDNF levels and the increase of p75NTR expression at both ages (n = 5). Comparable amounts of samples are loaded. Representative western blot of NGF, BDNF and p75NTR and its quantification in 1 month old mice (*P < 0.05) and 15 days old mice (*P < 0.05).

The subcellular localization of p53 seems to play an important role in the activity of the protein. Both, import into the nucleus and export to the cytoplasm, appear to be mediated by specific pathways that are associated with either the stabilization of the active protein or its inactivation by degradation. Nuclear localization of p53 was shown to be mediated by nuclear localization signals. We measured the content of p53 in the nuclear and cytoplasmic fractions of cortical neurons from P15 and P30 mice by western blot. p53 protein was mainly localized in the nuclear fractions (Figure 6A), at both stages, while little signal was detected in the cytoplasmic fraction. These preliminary results suggest the implication of laforin in the regulation of p53 subcellular localization, although this hypothesis has to be confirmed through further experiments.

### Levels of NGF, BDNF and the receptor p75NTR in the cerebral cortex of Laforin-deficient mice

**Figure 7:**
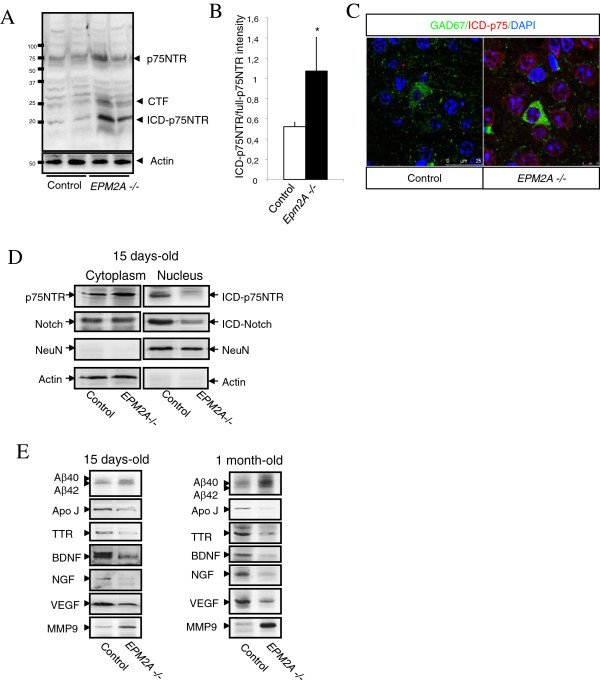
**Proteolytic processing of p75NTR in the cerebral cortex of *EPM2A*-/- mice at different ages. A)** Proteolytic processing of p75NTR (15 days of age). Representative western blot developed against cytoplasmic domain of p75NTR (ICD). The blot revealed fragments at 24 kDa consistent with the p75NTR-CTF, and a fragment at 19 kDa consistent with p75NTR-ICD, which was also weakly observed in the wild-type. We also detected an increase of p75NTR and ICD-p75 cleavage (*P < 0.05). Comparable amounts of samples are loaded, however α-actin was also used as a loading control. **B)** Quantification of the ratio ICD-p75NTR/ECD-p75NTR (15 days of age). **C)** Confocal images of ICD-p75NTR in GAD67 positive neurons **D)** There is not translocation of ICD units to nucleus in the cerebral cortex of mice with 15 days of age. We previously separated cytoplasm and nuclear fractions and we observed that there is not translocation of p75NTR-ICD and Notch-ICD to the nucleus. Representative blot of ICD units are shown (n = 5). As a loading control of cytoplasm fraction we used actin and as a loading control of nuclear fraction was used NeuN. **E)** Changes of soluble proteins in the cerebral cortex (n = 5). Low speed centrifugation of tissue extracts was used to isolate soluble fraction, which was analyzed by western blotting. These representative blots revealed that the main changes between wild-type and *EPM2A-/-* mice were noticed at 15 days-old and at 1 month-old. There was a significant decrease in the proteins implicated in neuroprotection and neurorepair: TTR, ApoJ, BDNF, NGF, VEGF; and there was a significant increase in proteins implicated with neuropathological situations: β-Amyloid 1-40 and 1-42 and MMP9. Results are mean ± SEM *p < 0.05, **p < 0.01 wild-type mice vs *EPM2A* -/- mice (ANOVA followed by Student’s *t* test).

Altered NGF and BDNF metabolism could be induced or exasperated by neurodegeneration in the brain of patients with Lafora disease. To characterize NGF and BDNF expression in cerebral cortex from *EPM2A*-/- mice, we carried out western blot analysis of these two proteins and the p75NTR, a receptor implicated in the apoptosis of neurons. For these experiments we used cerebral cortex lysates from 15 days and 1 month-old mice. At both ages NGF and BDNF levels decreased in *EPM2A*-/- mice, compared to wild type controls, while p75NTR increased (Figure 7A).

A relevant issue in the analysis of p75NTR signalling is also the evaluation of the presence or the accumulation of p75NTR intracellular domain ICD-p75NTR (20 KDa), which has recently been suggested to be involved in p75NTR signalling [24,25]. We described for the first time the pathophysiological role of p75NTR in Lafora disease and the possible increase in the ICD-p75NTR levels.

We used an antibody raised against the ICD-p75NTR (produced as described in the Methods section) to analyze cerebral cortex samples from *EPM2A*-/- mice at P5, P15 and P30. The antibody revealed the presence of two bands of 20-kDa and 25-kDa, respectively corresponding to the ICD-p75 and the carboxyl terminal fragment (CTF)-p75NTR [26,27], products of α- and γ-secretases activity. Both fragments appeared to be increased in the cerebral cortex of P15 *EPM2A*-/-, as shown by densitometry analysis of the 20-kDa band respect to total p75NTR signal revealed significant differences between the control and Laforin deficient brains in the cerebral cortex (Figure 7A, B and C).

The endogenous ICD-p75NTR was present in both the cytoplasmic and nuclear fraction of the control sample. However, we did not detect nuclear translocation of ICD-p75NTR fragment in the Laforin-deficient mice (Figure 7D). To assess the specificity of the fractionation procedure we checked the expression of NeuN, a nuclear protein, and a cytoplasmic actin in cellular lysates. Intramembrane cleavage events have been detected in many cell types, not only for p75NTR. To test whether the inhibition of nuclear translocation of ICD-p75 in the cerebral cortex of Laforin-deficient mice is specific or presenilin-dependent, we assessed also the ICD levels of Notch. We also confirmed that in *EPM2A*-/- mice ICD-Notch did not translocate to the nucleus (Figure 7D). This experiment upholds that in Lafora disease there is a possible impairment of the proteolysis through presenilin-dependent γ-secretase activity occuring at P15, before the development of LBs.

The activity of intramembrane proteolysis determines the release of soluble proteins in the milieu of cerebral cortex. We analyzed the soluble proteins pattern in *EPM2A-*/- mice cerebral cortex at P5, P15, and P30. Low speed centrifugation of tissue extracts (see Methods section) was used to isolate soluble fractions, which were analyzed by western blotting. The main alterations in *EPM2A-/-* mice were noticed at P15 (15 days-old) and P30 (1 month-old) (Figure 7E), revealing a significant decrease in proteins implicated in neuroprotection and neurorepair, such as transthyrretin (TTR), Clusterin (ApoJ), BDNF, NGF, VEGF) and a significant increase in proteins implicated in neuropathological conditions, such as β-Amyloid 1-40 and 1-42 and MMP9.

## Discussion

Mice with mutated *EPM2A* gene develop many of the characteristics of Lafora disease, since they show LBs in liver, muscle and brain, as well as impaired behavioural responses, ataxia and ultimate appearance of spontaneous myoclonic seizures [12]. Glycogen phosphate is already increased in *EPM2A*-/- at 3 months of age, and continued to rise over time [14]. LBs also appear around the third month [15], while the epilepsy progression starts at around 9 months of age [12]. Grand mal tonic-clonic seizures, which are almost invariably observed in Lafora disease patients, are clinically absent in 1 year-old mice [12], even in presence of LBs, suggesting that additional factors, environmental or genetic, are contributing to the pathophysiology of the disease. A possible interpretation of these differences could be related to the diversity in the functional organization of cortico-reticular-cortical pathways between mice and humans.

In the present work, we confirmed the presence of LBs in neurons of *EPM2A*-/- mice 3 and 13 months-old and we also determined, for the first time, that polyglucosan inclusions specifically affect GABAergic cortical neurons at early stages. The specific localization of the inclusions could be a key issue in interpreting the cerebral cortex dysfunction in Lafora disease. Pointing in this same direction, we also showed that the population of GABAergic cortical neurons is reduced in Laforin-deficient mice, which again underlie a possible specific role of GABAergic neurons in the development of the disease. Interestingly, autopsy of human patients showed that LBs are mainly abundant in layers III and V of the cortex, and other abnormalities were also noted in the pyramidal cells of the same layers [28]. The 15% of cortical neurons are local inhibitory interneurons, which are found in all cortical layers. Using the synapse category as a guide, it is estimated that 84% of all synaptic connections in the neocortex are excitatory and 16% are inhibitory [29]. EEG studies comparing Lafora disease with another progressive myoclonic epilepsy, Unverricht-Lundborg disease, have suggested that the sensory and motor cortices in Lafora disease are hyper-excitable in response to afferent stimuli [30,31]. Through unknown mechanisms, the inhibitory regulation of the cortex is impaired and seizures ensue. It is known that the electrophysiological profiles of these two diseases are quite distinct. When examining for motor evoked potential modulation by afferent sensory stimuli, there is early facilitation in Unverricht-Lundborg disease, whereas Lafora disease has delayed and prolonged facilitation [30].

At the same time we pretended to further characterize the cause of neuronal degeneration in Lafora disease, which could be due either to the presence of polyglucosan inclusions, or to some metabolic defect most likely related to laforin dysfunction. Neuronal cell death was described by Ganesh et al, 2002 at all stages of *EPM2A*-/- mice between 2 to 12 month-old animals [12]. However, a rigorous quantitative analysis for different age groups is necessary to understand the spatial and temporal difference in inclusions distribution to reveal the cause of degenerating neurons. The defining characteristics of apoptosis, the fragmentation of both cytoplasm and nucleus were not found in the degenerating neurons. We evaluated neuronal cell death by TUNEL technique and we could not find TUNEL positive cells, suggesting that cell death occurs without the activation of apoptosis. It is worth to point out that morphologically very similar features of cell death have been previously described for neuronal degeneration in Huntington disease, both in patients and mouse models [32].

To characterize the causes of neurodegeneration we evaluated the number of PAS positives LBs and ground-glass inclusions, in all brain regions of *EPM2A*-/- mice. We found that ground-glass inclusions localize preferentially in cerebral cortex and hippocampus (Additional file 2: Figure S2), with lower amounts in the basal forebrain and random distribution throughout the rest of the brain. Whether the ground glass inclusions are precursors of LBs is still uncertain, however it is possible that the formation of these inclusions could represent the first step of LBs formation in the cytoplasm. Recently, Delgado-Escueta’s group detected that cell death and LBs showed a progressive increment in size and number with age. They found that ground-glass inclusions emerged at 2 weeks-old of age and LBs appeared at 2 months-old of age [33]. Moreover, there are ultraestructural evidences of neuronal somatic degeneration in Purkinje cells, hippocampal pyramidal and granular cells, and cerebral cortical pyramidal cells in 2 months old mice in spite of the appearance of the first LBs at three months of age [12]. It is possible that the absence of laforin could induce a specific damage in GABAergic neurons previously to the appearance of first the LBs, killing the affected neurons just between P15 and P30. Alternatively, laforin could be the key factor in neuronal migration process, being involved in the maintenance of the adequate environmental conditions in cerebral cortex to induce GABAergic development. Indeed, it was published that Lafora disease is associated with a reduction in cortical glucose metabolic rate and cerebral blood flow and a lowered oxygen rate as determined by positron emission tomography [34]. Although we can not exclude either of the two hypotheses, we suggest that a correct balance between amounts of neurons of different type is necessary for correct brain functionality and that the proliferation of GABAergic neurons has to be equilibrated with the generation of glutamatergic neurons. Therefore we suggest that the imbalance between neuron populations in *EPM2A-/-* mice disturb the cortical network resulting in the pathogenesis of Lafora disease.

Further more, we described that of synaptophysin and synaptotagmin expression at early ages (since P5) is downregulated in the cerebral cortex of *EPM2A-/-* mice, indicating a reduction in the number of synapse. This evidence could suggest an important new role for laforin in the formation or the maintenance of synapses, which is worth to be more deeply investigated, in order to be confirmed.

The *EPM2A*-/- mice brain also show the proliferation of enlarged lysosomes, lipofuscin granules, β-amyloid peptides and increased levels of insoluble form of ubiquitinated protein, indicating a significant impairment in the cellular degradation pathway [35,36]. Moreover, abnormal dendrites and increased gliosis, especially in proximity of LBs, were noted in Lafora disease brain (Additional file 3: Figure S3). It is well described that in other neurodegenerative diseases, such as Alzheimer’s disease, neurotoxic peptides induce the activation of lysosomal pathways and the increase of the lysosomal marker LAMP2 levels [37]. LAMP2 acts as a receptor for a selective pathway of degradation of cytosolic proteins in lysosomes known as chaperone-mediated autophagy. In this pathway, specific cytosolic proteins are directly transported through the lysosomal membrane into the lysosomal matrix where they are degraded. Recently, it was suggested that some of the neuropathological changes in Lafora are likely to be side effects caused of the presence of LBs [36]. Moreover, it was demonstrated that impairment in the autophagy-endosomal-lysosomal pathways might underlie some of the symptoms in Lafora disease [36,38]. Analyzing the expression of LAMP2, we demonstrated that lysosomal activity and autophagy are increased at different ages in the cerebral cortex of *EPM2A*-/- mice, corroborating the results published by Puri et al, 2012 [36].

In addition, we highlight the presence of actin disarrangement in cortical neurons of *EPM2A*-/- mice, which resembles the characteristics of other neurodegenerative disease, such as Alzheimer’s disease, where actin deposits have been found in the hippocampus and the cerebral cortex of post-mortem brains, predominantly localized in amyloid containing neurites [39]. The origin and role of these inclusions in Lafora disease are unknown, however, this data do arise the possibility that LBs contribute to the actin disarrangement and could be involved in the loss of synapses.

Current clinical and neuropathological views consider LBs to be the cause of neurological derangement of patients, however the defining characteristics of apoptosis in neurons were not found in *EPM2A*-/- mice. During the last year, Machado-Salas et al. described the presence of necrotic neuronal death in the hindbrain, in absence of LBs, in young *EPM2A*-/- mice (P11) [33,40]. They demonstrated that both cell death and LBs showed a increase progressively with age.

In the present article, we show an increase of active-caspase-3 in the cerebral cortex of 1 month-old mice, which surprisingly appears specifically in the glutamatergic neurons of the cerebral cortex. We could not detect any GABAergic neuron with active-caspase-3 or TUNEL-positive staining at any of the three ages, suggesting that the decrease of GABAergic neurons population is not due to specific cell death, but it is rather probable that the lack of laforin induces an impaired GABAergic neurodevelopment in the cerebral cortex.

In order to evaluate the effects of the lack of laforin in the development of the cerebral cortex of *EPM2A*-/- mice, we detected the content of p53 in subcellular fractions of cortical neurons from P15 and P30 mice. The tumour suppressor gene p53 plays a central role in the maintenance of genomic stability [41,42]. Versatility in p53 activity, as a response to various external or internal signals, may serve as a control mechanism, which underlies central decisions in developmental pathways *in vivo*[43]. Furthermore, a number of studies indicate the involvement of p53 in developmental pathways of neural cells. Differentiation of rat primary cultures of neurons in culture was shown to be accompanied by the migration of p53 protein into the nuclear compartment [44]. The subcellular localization of p53 seems to play an important role in the activity of the p53 molecule. Both, import into the nucleus and export to the cytoplasm, appear to be mediated by specific pathways that are associated with either the stabilization of the active protein or its inactivation by degradation, respectively. p53 was detected in the nuclear fraction of P15 and P30 mice, while little signal was detected in the cytoplasmic fraction. Although more experiments are necessary, these preliminary results suggested that laforin is related to the regulation of p53 subcellular localization.

It is beyond doubt that the neurotrophin family of proteins plays key roles in determining the fate of the neuron, not only during embryonic development, but also in the adult brain [45]. Neurotrophins such as NGF (nerve growth factor) and BDNF (brain-derived neurotrophic factor) can play dual roles: first, in neuronal survival and death, and, secondly, in activity-dependent plasticity. BDNF is abundantly expressed in the brain and plays a crucial role in activity-dependent plastic changes in synaptic strength and network refinement [46,47]. BDNF has been implicated in regulating adult neurogenesis in the subventricular zone of the dentate gyrus and also in the development of the GABAergic interneurons [48,49]. BDNF also has been described in the epilepsy [50,51]. The implications of neurotrophins in the development of Lafora disease are unknown. We hypothesize that, since Lafora disease is a type of epilepsy with GABA neurons impairment, it is possible that neurotrophins may be involved in disease development. The neurotrophins manifest their effects by binding to two discrete receptor subtypes: the Trk (tropomyosin receptor kinase) family of RTKs (receptor tyrosine kinases) and the receptor p75NTR [26]. The altered metabolism of NGF and BDNF could be induced or aggravated by the neurodegeneration in the brain patients with Lafora disease. To characterize the NGF and BDNF expression in cerebral cortex from *EPM2A*-/- mice, we evaluated analysis of NGF, BDNF and the receptor implicated in the apoptosis of neurons, the p75NTR in cerebral cortex lysates from 15 days-old and 1 month-old of age. At both ages NGF and BDNF levels decreased and p75NTR increased. The receptor p75NTR is known to be involved in signalling apoptosis in a number of cell models by NGF binding [52,53]. The increase of p75NTR in Alzheimer’s disease affected human brain is not fully accepted in the literature, nonetheless the increase of p75NTR processing, which tends to translocate to the nucleus, was described in this neurodegenerative disease [54]. We described for the first time the pathophysiological role of p75NTR in Lafora disease; we showed an increase in the ICD-p75NTR levels. Whether the ICD-p75NTR is directed to the nucleus has been very difficult to determine due to the instability and the extremely low levels of the ICD fragment [55]. Notwithstanding, in our study it remains to be established whether the ICD-p75NTR generated is directed to the nucleus of these neurons. Our data revealed a surprising result in laforin deficient mice, since we were not able to detect nuclear translocation of ICD-p75NTR fragment. Intramembrane cleavage events have been detected not only for p75NTR in many cell types [56]. Proteolysis through presenilin-dependent γ-secretase activity has emerged as a highly conserved and prevalent mechanism in receptor signalling responsible for the intramembrane cleavage of important proteins, such as Notch, ErbB4 tyrosine kinase receptors, CD44, low density lipoprotein, and β-amyloid precursor protein [57]. To test whether this inhibition of nuclear translocation of ICD-p75 in the cerebral cortex of Laforin-deficient mice is specific or presenilin-dependent, we assessed also the ICD levels of Notch, confirming that in *EPM2A*-/- mice ICD-Notch did not translocate to the nucleus. This experiment uphold that in Lafora disease there is a signalling alteration in the proteolysis through presenilin-dependent γ-secretase activity and this process occurrs at the age of 15 days-old, before the development of the first LBs.

Up to date, no information regarding p75NTR processing or nuclear translocation of ICD-p75 in apoptosis is available. Several years ago, a first physiological role was detected for ICD-p75 yield under myelin-associated glycoprotein activation in cerebellar neurons, where the down-regulation of TACE by RNA interference blocked BDNF-induced p75NTR cleavage and apoptosis [25,54]. However, our results suggest that impairment of p75NTR signalling and altered neurotrophins levels (NGF and BDNF) could be involved in the neurodegenerative processes of Lafora disease in the cerebral cortex. Our data prompt that p75NTR may employ regulated intramembrane proteolysis to transmit an intracellular signal. Similar to the cleavage of Notch, the ICD-p75NTR may function as a nuclear transcriptional modulator, activating/deactivating a set of genes that are involved in cell death or neuronal differentiation. The relationship between this process and the induction of neuronal apoptosis is still not completely understood, nevertheless these changes in the neurotrophin signalling occurred before the appearance of the first LBs. Polyglucosan bodies are known to be produced normally in neuronal cell and cleared via axons into cerebrospinal fluid [58]. A physiological role described for ICD-p75NTR was under myelin-associated glycoprotein activation in cortical neurons for activation of Rho and inhibition or neurite outgrowth [59]. These results come up with laforin and p75NTR signalling could be involved in the intercellular and intracellular migration of polyglucosan, and its loss of function would result in polyglucosan accumulation as LBs.

Lafora disease has been previously associated altered protein clearance [60,61]. We believe it is crucial to analyze the release of soluble proteins, related with the intramembrane proteolysis, in the cerebral cortex in *EPM2A-*/- mice, prior to the appearance of the first LBs. Our findings showed that there was a significant decrease in the proteins implicated in neuroprotection and neurorepair: transthyrretin (TTR), Clusterin (ApoJ), BDNF, NGF, VEGF; and there was a significant increase in proteins implicated with neuropathological situations: β-Amyloid 1-40 and 1-42 and MMP9. It was previously published that the level of the Ser^9^-phospho (inactive) form of GSK3β was lower in the 10 month-old *EPM2A*-/- mice compared to controls, suggesting that laforin probably acts upstream of this key enzyme [35]. AKT, PKA, and PP1 are a few of the known regulators of the Ser^9^ residue of GSK3β [62,63], however, none of the three showed a significant change in its phosphorylation pattern, as analyzed by immunoblot of neuronal lysates from *EPM2A*-/- mice with 10 month-old.

## Conclusions

Our results suggest that Lafora disease is mainly a neurodegenerative disease, since neurodevelopment impairment develops specifically in the cerebral cortex prior to LBs formation. It is likely therefore that LBs and neurological damage are independent consequences of the same defect in a common physiological pathway and it is possible that only some Lafora disease symptoms are a consequence of LBs formation. Because Lafora disease has been traditionally classified as a glycogen metabolism disorder, only a few reports described the neurodegenerative changes in the neuropile [3,11,64-66]. Nevertheless, our data strongly support the idea that Lafora disease is both a complex neurodegenerative disease and a glycogen metabolism disorder.

The basis that we set up through our findings, are critical for future studies on the pathology in the cerebral cortex and hippocampus, as well as for the development of new therapies for Lafora disease. Interestingly, the neurodegenerative changes observed in this Lafora disease model can also be useful for understanding the neurotrophin signalling in the process of dementia.

## Methods

### Animals

Brain tissues of laforin-deficient mice (*EPM2A*-/- mice) and their wild-type littermates [12] obtained in frozen condition and fixed in paraformaldehide from Dr. Santiago Rodríguez de Cordoba (The Centro de Investigaciones Biológicas, Spanish National research Council, CSIC, Madrid, Spain) were homogenized and processed for immunoblotting as reported earlier. Cerebral cortex from wild-type and *EPM2A*-/- mice of the same age (15 days-old, 1 month-old, 3 months-old and 13 months-old) were isolated in ice-cold lysis buffer and processed following different protocols for total lysates and subcellular fractioning [27]. We analysed samples by immunofluorescence coupled to confocal microscopy and immunoblot assays. All animals were handled and cared for in accordance with European Community Council Directive (86/609/EEC).

### Animal ethics statement

All of the experiments were performed according to ethical regulations on the use and welfare of experimental animals of the European Union and the Spanish Ministry of Agriculture, and the procedures were approved by the bioethical committee of the University Hospital of Vigo.

### Immunoassays

For Western blot analysis brain tissue samples were lysed in PIK buffer (150 mM NaCl, 20 mM TrisHCl pH 7.4, 1% (v/v) NP40 and protease inhibitors: 1 μg/mL aprotinin, 1 μg/mL leupeptin and 1 μg/mL phenylmethylsulfonyl fluoride, PMSF). For cell fractionation cells were lysed with C buffer (10 mM HEPES, 60 mM KCl, 1 mM EDTA, 0.075% (v/v) Triton X100, 1 mM DTT with protease inhibitors). The pellet nuclei was obtained by centrifugation 325 g, 4 minutes and resuspended in NB buffer (20 mM TrisHCl pH 8, 420 mM NaCl, 15 mM MgCl2, 0.2 mM EDTA, 25% (v/v) Glicerol and protease inhibitors). Thereafter, the nuclei supernatant were obtained after centrifugation 9000 g, 10 minutes [67]. To separate the soluble and insoluble fractions, the brain was lysed and homogenized in PBS, and centrifuged at 10.000 g. After running the samples in acrylamide gels, proteins were transferred (immobilon, Bio-Rad) and membranes were incubated with the corresponding primary antibodies at 4°C overnight. Afterwards, membranes were washed and incubated with secondary antibodies. Membranes were washed several times with Tween-TBS and developed with ECL plus (Amersham). Western blot membranes were re-blotted with unrelated proteins (actin and βIII-Tubulin)) as an internal standard and normalized for protein load. Densitometric analysis was performed using ImageJ software (NIH Image). A representative blot is shown from a total of at lest three independent experiments.

### Immunofluorescence

The brain was immersion fixed in paraformaldehide 4% (w/v) in PBS 0.1 M (NaH_2_PO_4_ *H_2_O (13.8 g/L), Na_2_HPO_4_ (14.2 g/L)_,_ NaCl (8 g/L), pH 7.4) for 24 h, paraffin embedded sections. Sections (10 μm thick) of the brain were then cut using a microtome (Leica Microsystems, UK). Brain sections were blocked with 5% (w/v) BSA and incubated overnight at 4°C with the respective antibody in PB containing 0.5% (w/v) BSA and 0.1 (v/v) Triton X100. After several washes in PB, sections were incubated with Alexa-coupled secondary antibody in the same PB buffer. Emission of primary antibody was used as control. Confocal analysis was performed in a Leica confocal microscope. For immunohistochemical analysis, preparations were visualized by light microscopy using DAB conjugated avidin-biotin complex kit (Vectastain ABC Elite, Vector Laboratories, USA). For periodic acid-Schiff (PAS)-Diastase staining, sections were incubated with diastase prior to staining with PAS reagent for detection of Lafora bodies.

### Fluorescence microscopy analysis of Rhodamine Phalloidin-stained cerebral cortex

Slices were washed twice with phosphate-buffered saline, fixed in 4% (w/v) paraformaldehyde solution for 10 min at room temperature, permeated with 0.1% (v/v) Triton X-100, and stained with rhodamine-phalloidin. Fluorescent images were monitored using a confocal microscopy located in the “Centro de Apoio Científico-Tecnolóxico a Investigación, CACTI, University of Vigo”.

### Quantification of cell death by TUNEL staining

Apoptotic cells were detected using an apoptosis in situ cell death detection kit (Roche). Analysis of apoptotic cells was based on the TUNEL procedure, which consists of the addition of apoptotically fragmented DNA to the 3′ termini by terminal deoxynucleotidyl transferase followed by immunochemical detection using an anti-fluorescein antibody conjugated with horseradish peroxidase and diaminobenzidine as a substrate.

### Antibodies

The following antibodies were used: rabbit polyclonal anti-p75NTR against extracellular domain (9651) and intracellular region (9992) gifted from Dr. M. Chao and Dr. B. Carter [68]. Mouse monoclonal anti-α-actin (Sigma), mouse monoclonal anti-βIII-Tubulin (Millipore), anti-polyglucosans (Kamiya Biomedical Company, USA), polyclonal anti-GAD67 (Abcam), Mouse monoclonal anti-synaptophysin (Chemicon), Mouse monoclonal anti-synaptotagmin (Sigma), mouse monoclonal anti-β-amyloid (MBL), mouse monoclonal anti-caspase-3 and rabbit anti-caspase-3-active (Cell Signalling), mouse monoclonal anti-caspase-9-active (Cell Signalling), mouse monoclonal anti-p53 (Dako), mouse-monoclonal anti-NeuN (Millipore), Mouse monoclonal anti-LAMP2 (Abcam), polyclonal anti-LC3 (Cell Signalling), polyclonal anti-NGF (Chemicon), polyclonal anti-BDNF (Abcam), goat-polyclonal anti-Notch (Santa Cruz Biotechnologies), rabbit polyclonal anti-Transtyrretin (Santa Cruz Biotechnologies), polyclonal anti-Apolipoprotein J (Abcam), polyclonal anti-VEGF (Santa Cruz Biotechnologies), polyclonal anti-MMP9 (Santa Cruz Biotechnologies), rabbit polyclonal anti-phospho Akt (Cell Signalling), rabbit polyclonal anti-phospho JNK (Cell Signalling), rabbit polyclonal anti-Akt (Cell Signalling), mouse monoclonal anti-AT8 (Termo Scientific), phalloidin-Rhodamin (Invitrogen). All Alexa-fluor antibodies were purchased from Invitrogen.

### Cell counting

Stereological cell counts and measurements were carried out using microscope with digital camera and software for morphometry (Leica AMD). GAD67 neurons cell number in the cerebral cortex was estimated using standard model-based stereological methods [69]. Briefly, for neuronal counts, brains were blocked and serially-sectioned at 10 μm from the frontal brain to the cerebellar-midbrain junction. Serial sections were mounted 5 sections per slide onto polyionic slides. GAD67-positive neurons were counted using a 40× objective (total magnification 400×). Specifically, neurons from both left and right sides of the cerebral cortex within one section per slide (chosen randomly and then maintained throughout all sections were counted). A neuron was considered to be immunopositive if it was labelled by the typical brown colour of the DAB peroxidase reaction product in the cytoplasm and proximal dendrites (see Additional file 1: Figure S1A).

## Abbreviations

CTF: Carboxyl terminal fragment; ICD: Intracellular domain; GABA: Gamma aminobutyric acic; LB: Lafora body; MMP: Metalloproteinase; RTKs: Receptor tyrosine kinases.

## Competing interests

None of the authors of this manuscript have any financial interest that has influenced the results or interpretation of this manuscript.

## Authors’ contributions

The work presented here was carried out in collaboration between all authors. SO and CS defined the research theme, CS, IV and RCAB elaborated the animal processing and experiments. SO and CS analyzed the data and interpreted the results. CS and SO wrote the paper. All authors have contributed to see and approve the manuscript.

## Supplementary Material

Additional file 1: Figure S1 The pathological hallmark of Lafora disease is the presence of cytoplasmic LBs in neurons. The LBs are indicated with arrows in the picture. A) Immunostaining with the neuronal marker βIII-Tubulin in temporal and frontal cortex sections of *EPM2A*-/- mice at different ages: 3 and 13 months-old (upper pictures) and combined with PAS staining for specific detection of the LBs (bottom pictures). B) Confocal images of sections of cerebral cortex stained with cortical neurons marker GAD67 (green) and LBs detected with specific polyglucosan antibody (red). Click here for file

Additional file 2: Figure S2 Detailed analysis of LBs located in the cytoplasm and dendrites. A) Stereological cytoplasm LBs and ground-glass inclusions (LBs located in dendrites) counted and measured with microscope with digital camera and software for morphometry in *EPM2A*-/- mice at 5 months-old (n = 3). Although the LBs and ground-glass inclusions appear in all brain regions, at early ages the highest density was placed in cerebral cortex and hippocampus, with lesser amounts in the basal forebrain and sparse distribution through out the rest of the brain. B) Cytoplasm LBs emerged at 3 months of age and LBs in dendrites appeared around 15 days of age. This schematic representation suggests that the temporary window where the molecular changes occur before the first LBs are located in the neurons is around 1moth-old. Click here for file

Additional file 3: Figure S3 Reactive gliosis in the brain temporal cortex (3 month-old) in *EPM2A*-/- mice in paraffin blocks (see Methods) and developed with DAB. A) GFAP immunostaining (left) and PAS staining (right) of the same cortical area in Laforin-deficient mice. B) Broad image of GFAP immunostaining combined with PAS staining of cerebral cortex at *EPM2A-*/- mice. Click here for file
