# Supplementary material for: Loss of GABAergic cortical neurons underlies the neuropathology of Lafora disease
Source: Mol Brain. 2014 Jan 28;7:7. doi: 10.1186/1756-6606-7-7 (PMC3917365; doi:10.1186/1756-6606-7-7)
Supplement: Additional file 1: Figure S1 — The pathological hallmark of Lafora disease is the presence of cytoplasmic LBs in neurons. The LBs are indicated with arrows in the picture. A) Immunostaining with the neuronal marker βIII-Tubulin in temporal and frontal cortex sections of EPM2A-/- mice at different ages: 3 and 13 months-old (upper pictures) and combined with PAS staining for specific detection of the LBs (bottom pictures). B) Confocal images of sections of cerebral cortex stained with cortical neurons marker GAD67 (green) and LBs detected with specific polyglucosan antibody (red). [file 1756-6606-7-7-S1.ppt]

## Slide 1
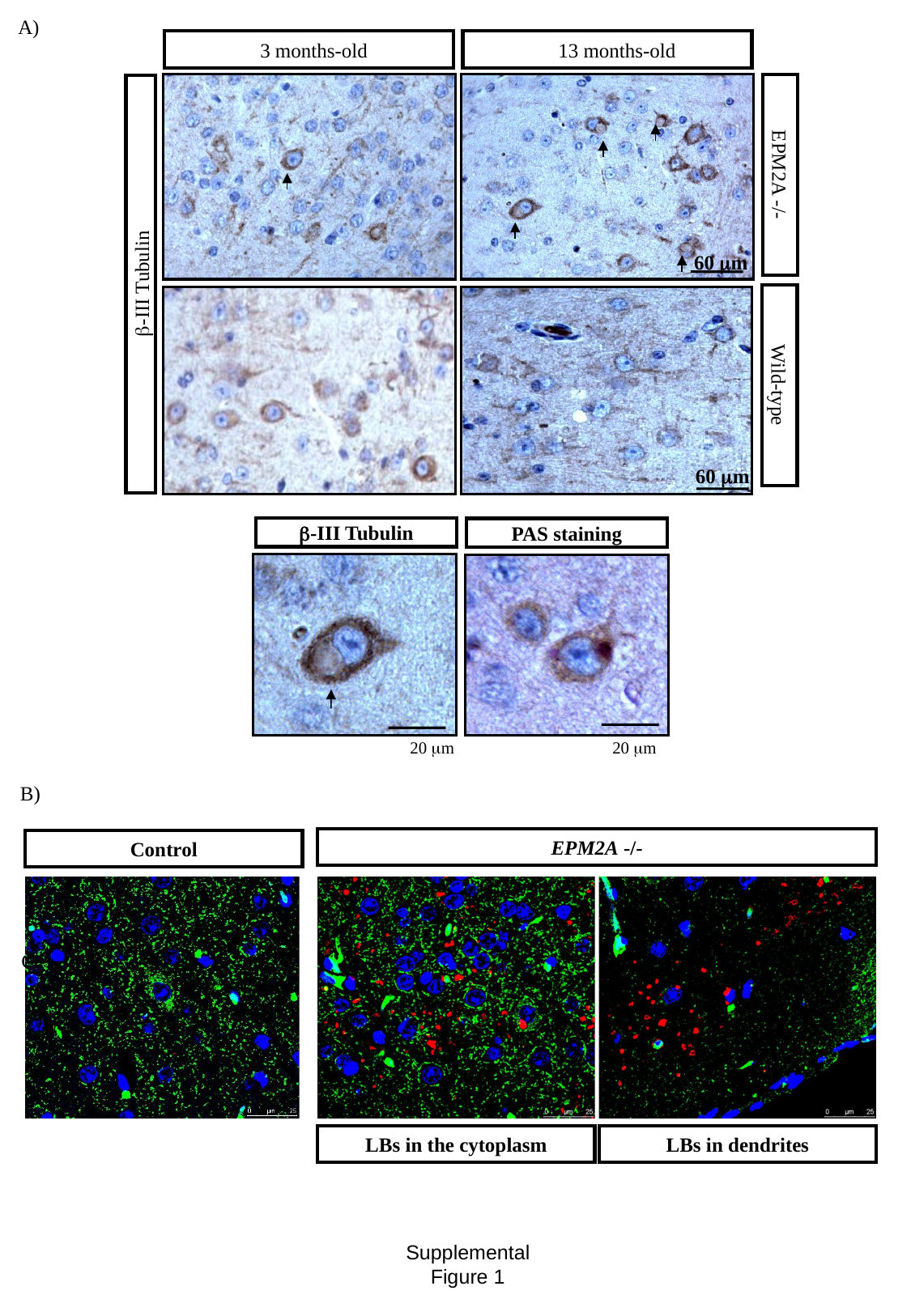

A)
3 months-old
13 months-old
EPM2A -/-
60 μm
β-III Tubulin
Wild-type
60 μm
β-III Tubulin
PAS staining
20 μm
20 μm
B)
EPM2A -/-
Control
C)
LBs in the cytoplasm
LBs in dendrites
Supplemental Figure 1
