# Supplementary material for: Loss of GABAergic cortical neurons underlies the neuropathology of Lafora disease
Source: Mol Brain. 2014 Jan 28;7:7. doi: 10.1186/1756-6606-7-7 (PMC3917365; doi:10.1186/1756-6606-7-7)
Supplement: Additional file 2: Figure S2 — Detailed analysis of LBs located in the cytoplasm and dendrites. A) Stereological cytoplasm LBs and ground-glass inclusions (LBs located in dendrites) counted and measured with microscope with digital camera and software for morphometry in EPM2A-/- mice at 5 months-old (n = 3). Although the LBs and ground-glass inclusions appear in all brain regions, at early ages the highest density was placed in cerebral cortex and hippocampus, with lesser amounts in the basal forebrain and sparse distribution through out the rest of the brain. B) Cytoplasm LBs emerged at 3 months of age and LBs in dendrites appeared around 15 days of age. This schematic representation suggests that the temporary window where the molecular changes occur before the first LBs are located in the neurons is around 1moth-old. [file 1756-6606-7-7-S2.ppt]

## Slide 1
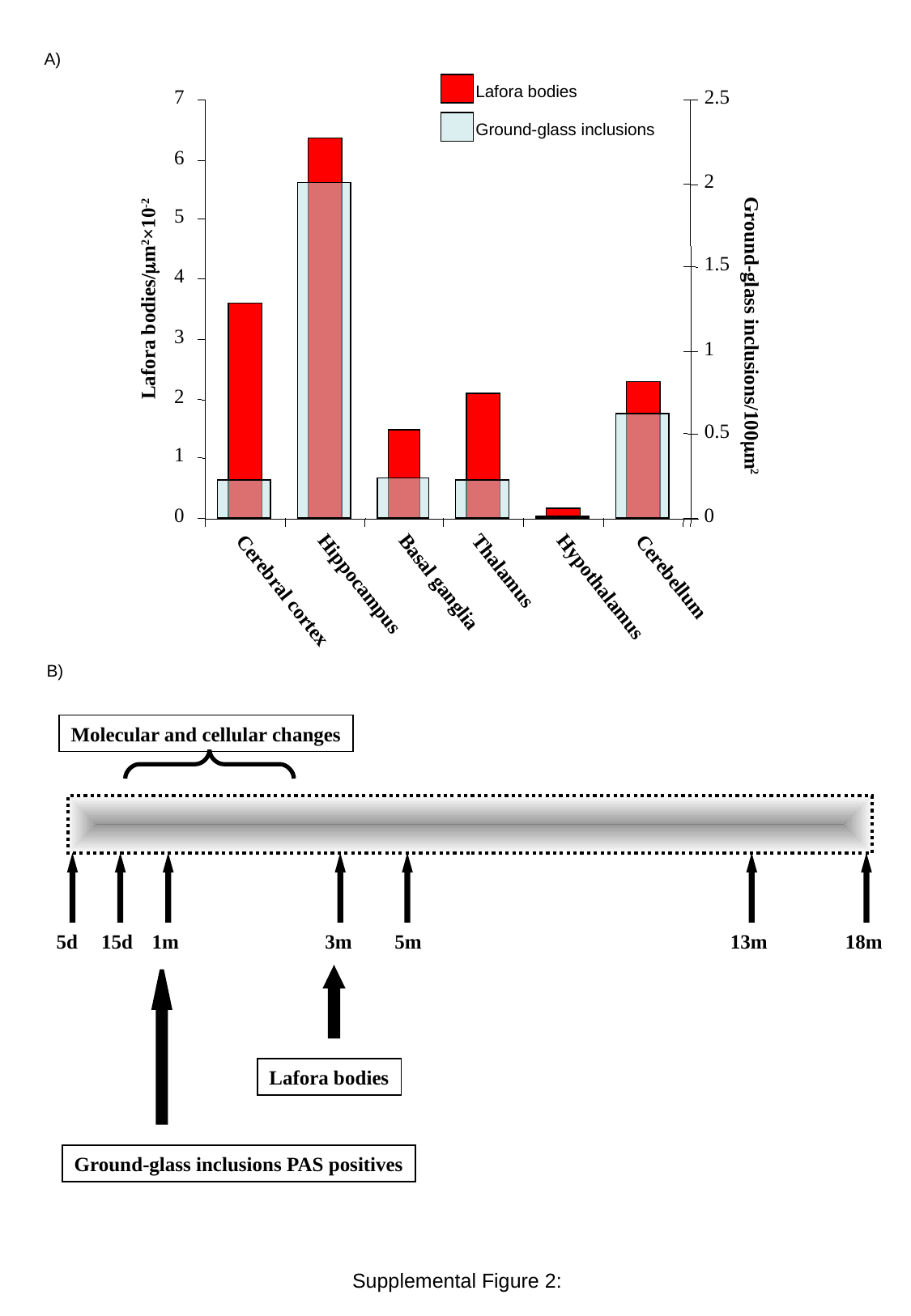

A)
Lafora bodies
Ground-glass inclusions
7
2.5
2
1.5
Ground-glass inclusions/100μm2
1
0.5
0
6
5
4
Lafora bodies/μm2×10-2
3
2
1
0
Thalamus
Cerebellum
Basal ganglia
Hippocampus
Hypothalamus
Cerebral cortex
B)
Molecular and cellular changes
5d
15d
1m
3m
5m
13m
18m
Lafora bodies
Ground-glass inclusions PAS positives
Supplemental Figure 2:
