# Supplementary figures and images for: Loss of GABAergic cortical neurons underlies the neuropathology of Lafora disease
Source: Mol Brain. 2014 Jan 28;7:7. doi: 10.1186/1756-6606-7-7 (PMC3917365; doi:10.1186/1756-6606-7-7)

## Slide 1
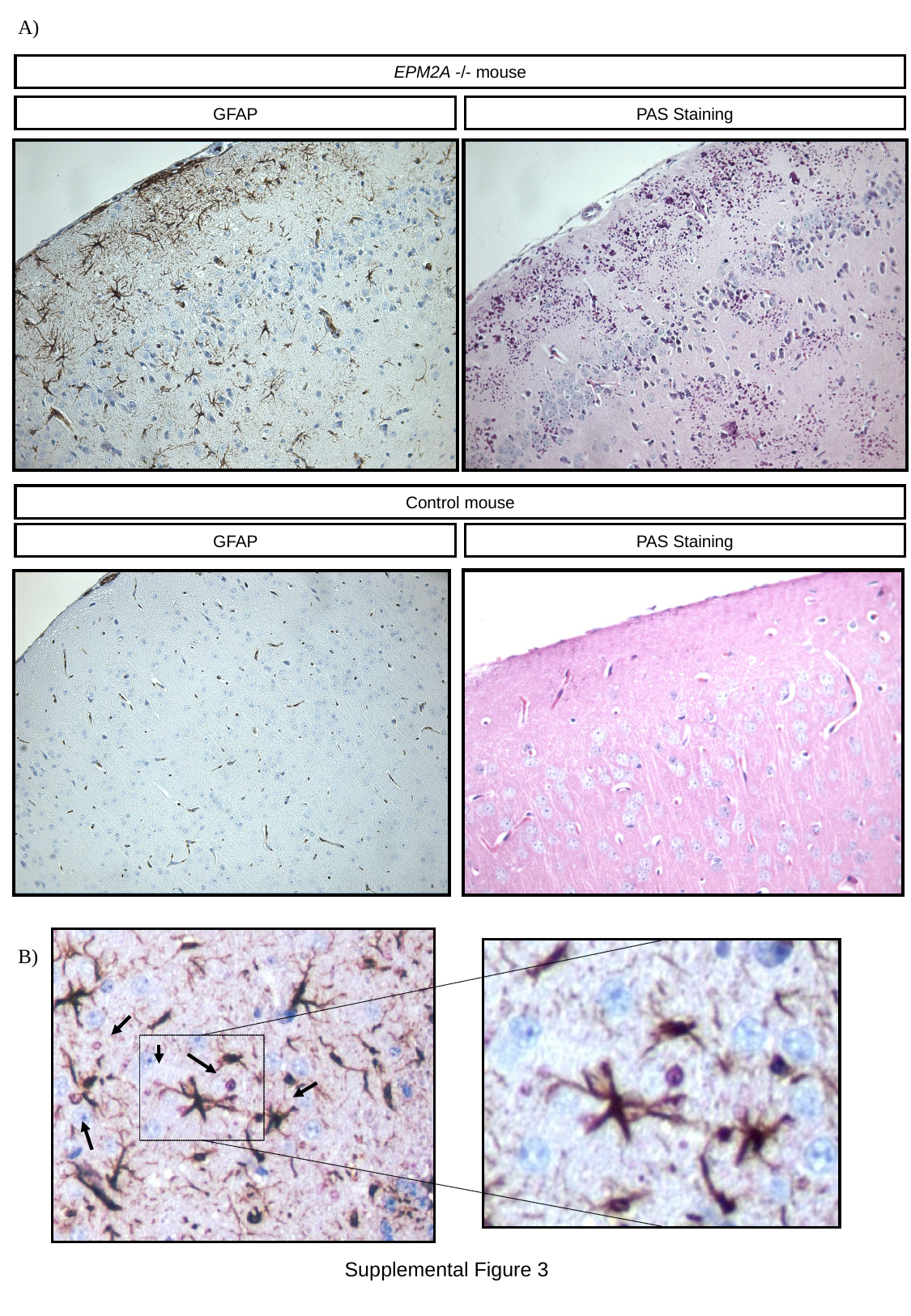

A)
EPM2A -/- mouse
GFAP
PAS Staining
Control mouse
GFAP
PAS Staining
B)
Supplemental Figure 3

Supplement: Additional file 3: Figure S3 — Reactive gliosis in the brain temporal cortex (3 month-old) in EPM2A-/- mice in paraffin blocks (see Methods) and developed with DAB. A) GFAP immunostaining (left) and PAS staining (right) of the same cortical area in Laforin-deficient mice. B) Broad image of GFAP immunostaining combined with PAS staining of cerebral cortex at EPM2A-/- mice. [file 1756-6606-7-7-S3.ppt]
